# Rare case of urethral catheter knot formation in a woman during elective surgery

**DOI:** 10.1186/s40981-022-00587-4

**Published:** 2022-12-09

**Authors:** Ayumi Oishi, Midori Mogami, Nobuhiro Kushida, Chiaki Nemoto, Satoki Inoue

**Affiliations:** 1 The Junior Resident Center, Ohara General Hospital, 6-1 Ohomachi, Fukushima, 960-8611 Japan; 2 Department of Anesthesiology, Ohara General Hospital, 6-1 Ohomachi, Fukushima, 960-8611 Japan; 3 Department of Urology, Ohara General Hospital, 6-1 Ohomachi, Fukushima, 960-8611 Japan; 4 Department of Anesthesiology, Fukushima Medical University, 1 Hikarigaoka, Fukushima, Fukushima 960-1295 Japan

**Keywords:** Urethral catheter, Knot formation

## To the Editor

Catheter-related accidental knot formation is rare but sometimes occurs when using central venous lines, epidural catheters, and nasogastric tubes [1–3]. Urethral catheters are not an exception. Causes of urethral catheter knot formation include the insertion length, size, and elasticity of the catheter as well as the operator’s skills. Most cases of urethral catheter knot formation occur in children or infants because of the small diameter of the catheter for its length and the soft elasticity [4]. In adult patients, knot formation is theoretically very rare because the diameter of the catheter is large for its length, and the catheter is comparatively rigid. We experienced a case of urinary catheter knot formation during elective surgery.

**Fig. 1:**
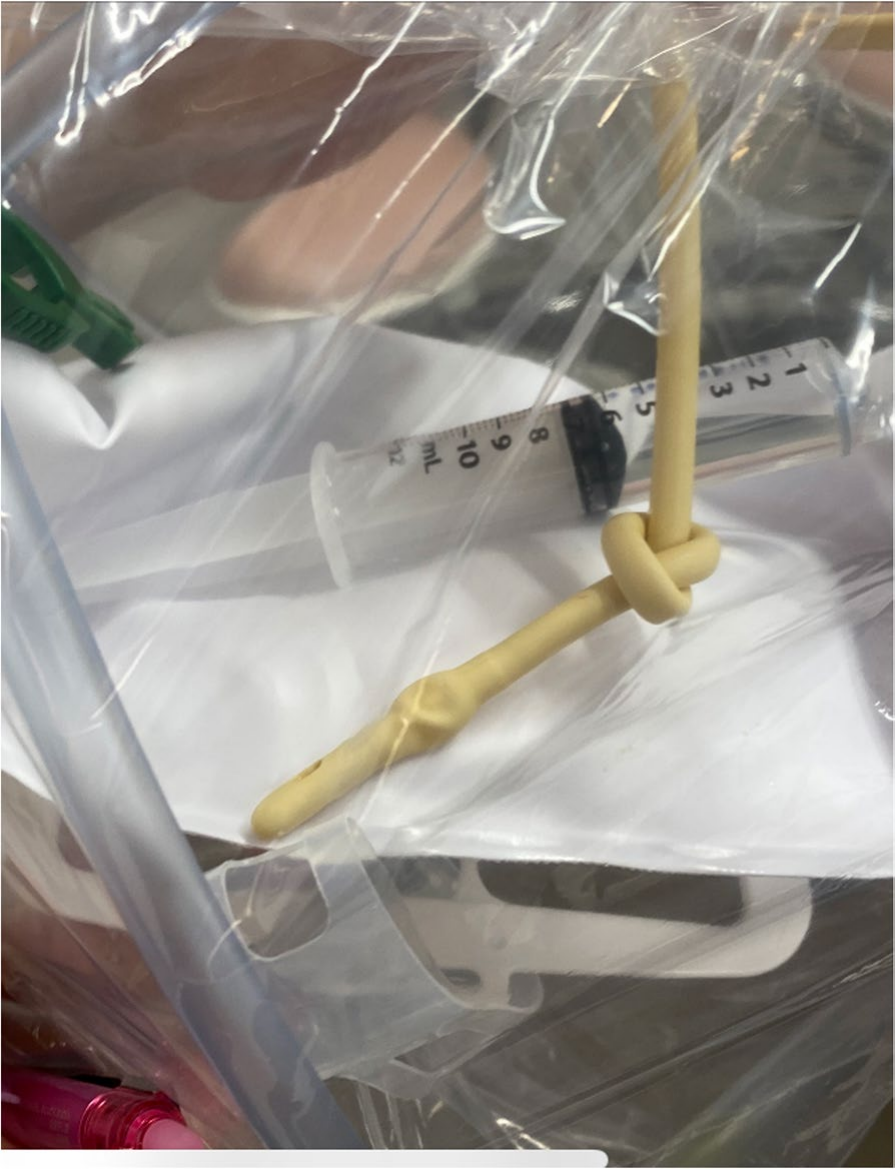
Photograph showing the removed urinary catheter after incomplete deflation of the balloon. The urinary catheter was removed manually after withdrawing 7 of 10 mL of distilled water in the balloon on the third postoperative day. A knot developed 6 cm from the tip of the catheter. The knot likely formed when the Foley catheter was inserted further to confirm urine outflow, at which time the catheter coiled back onto itself. The catheter was radiolucent; therefore, we could not identify the knot until the catheter was removed

An 88-year-old woman (height, 143 cm; weight, 51 kg) was scheduled for laparoscopic surgery. Her comorbidities did not include urologic disease. After induction of general anesthesia, a 14-Fr Foley urethral catheter was inserted without difficulty. Although the catheter was inserted deeply enough for a female patient, little urine outflow was observed. The balloon of the catheter was inflated with 10 mL of distilled water, and the catheter was withdrawn until resistance was felt. The operation was completed without difficulty, and no malfunction of the urethral catheter was observed perioperatively. On postoperative day 3, the urethral catheter was removed as planned. The nurse in charge of the patient experienced slight difficulty in deflating the balloon, but 7 of 10 mL of distilled water was withdrawn; therefore, the nurse removed the catheter. The catheter was withdrawn with slight resistance, and a knot was found in the catheter (Fig. 1). Fortunately, no urethral injury occurred, and the patient was discharged from the hospital on postoperative day 10.

Jallad et al. [5] reported that a 12-Fr Foley urethral catheter became knotted in a female adult patient. Mulawkar [6] reported knot formation in a 14-Fr straight urethral catheter during self-catheterization in a female adult patient. In both case reports, the authors suggested that the passage of an excessively long catheter might have caused catheter coiling and subsequent knotting in these female patients [5, 6]. In women, it is unnecessary to fully insert the urethral catheter because the average length of the female urethra is 4 cm [5]. Therefore, we can usually confirm urine outflow within that depth. However, in some cases, urine outflow cannot be confirmed after the insertion of the urethral catheter. Before advancing a catheter further to confirm urine outflow, we would consider point-of-care ultrasonography to identify a Foley catheter in the bladder. As in our case, in which the catheter was radiolucent, point-of-care ultrasonography might be useful to confirm knot formation if the catheter is difficult to remove. In our case, it was possible to remove the catheter even with knot formation. However, some urethral catheter knots require cystoscopy to puncture the balloon or to permit the use of a holmium laser to ablate the knot to achieve catheter removal [5, 6]. Thus, clinicians should be aware that female patients may be at the highest risk for urethral catheter knot formation.

## Data Availability

Not applicable.
